# Data on fate and distribution of organophosphate esters in the soil - sediments from Kathmandu Valley, Nepal

**DOI:** 10.1016/j.dib.2019.104822

**Published:** 2019-11-16

**Authors:** Ishwar Chandra Yadav, Ningombam Linthoingambi Devi

**Affiliations:** a Department of International Environmental and Agricultural Science (IEAS), Tokyo University of Agriculture and Technology (TUAT), 3-5-8, Saiwai-Cho, Fuchu-Shi, Tokyo, 1838509, Japan; b Department of Environmental Science, Central University of South Bihar, SH-7, Gaya-Panchanpur, Post-Fatehpur, P.S-Tekari, District-Gaya, 824236, Bihar, India

**Keywords:** *Kathmandu*, *Bagmati river*, *Sediments*, *Surface soil*, *Flame retardants*, *Organic pollutants*

## Abstract

Globally, soil and sediments are known as the likely sinks of various organic pollutants, such as organophosphate esters (OPEs). However, the fate of OPEs in soil/sediment matrices is limited in the whole of South Asia, especially if there should be an occurrence of Nepal. This data article elucidates the fate and distribution of OPEs in soil and sediment samples from the capital city of Nepal (Kathmandu). A total of eight different compounds of OPE was measured in soil (N = 19) and sediment (N = 20) samples collected during October 2014. The median concentration and composition of the individual OPE have been discussed. Additionally, health risk exposure due to ingestion and dermal contact of OPE was assessed to mark the endanger of OPE. Moreover, risk quotient (RQ) for fish, Daphnia, and algae was calculated to forecast the risk of OPEs on aquatic organisms.

**Table:** Specifications Table

Subject area	Environmental Science
More specific subject area	Geochemistry and Ecotoxicology
Type of data	Table, graph, figure
How data was acquired	Gas chromatography coupled with mass spectrometry, MS Excel (2016), ISBN SPSS (version 21)
Data format	Raw and analyzed data
Parameters for data collection	The surface soil and sediment samples were Freeze-dried, ground, and sieved with a mesh size of 500 μm.
Description of data collection	Surface soil and sediment samples were collected from the Kathmandu valley to investigate the fate and distribution of organophosphate ester flame retardants. Both soil and sediment samples were extracted, purified, and analyzed with Gas Chromatography attached with Mass Spectrometry.
Data source location	Nepal: Kathmandu (KTM),
Data accessibility	Data is given in this article
Related research article	Yadav, I·C., Devi, N.L., Li, J., Zhang, G., Covaci, A. 2018. Concentration and spatial distribution of organophosphate esters in the soil-sediment profile of Kathmandu Valley, Nepal: Implication for risk assessment. Science of the Total Environment 613–614C, 502–512 [1]

**Table:** 

**Value of the Data** • This data insight the contamination level of OPEs in soil and sediment of Kathmandu valley • The profiling data of OPEs will highlight the contribution of individual OPE in Nepalese environment • The health risk exposure assessment of OPEs indicated the danger level in the Nepalese population • This data will be useful for policymaker, stakeholder or government official to implement the environmental management plan • This data will also serve as the primary data for the researcher who wants to explore more about the fate of OPEs

## 1 Data

The concentration data of OPEs in surface soil and sediment samples are given in Table 5 and Table 6, respectively. The health risk exposure of OPEs via soil ingestion and dermal contact has been discussed in Table 6, Table 7, respectively. Table 8, Table 9, Table 10 describes the ecological risk quotient of OPEs to fish, daphnia, and algae in Bagmati River. Fig. 2 illustrates the site-specific profile of individual OPEs measured in soil and sediment samples. The spatial distribution of OPEs in soil and sediments has been described in Fig. 3.

**Table 1:** Detail of soil sampling.

Sample ID	Sampling site location	Lat & Long	Elevation (m)	Period of sampling	Avg temp	Avg wind speed	Avg rainfall	Remarks
KTM01	Sanepa	27°41′3.30″N 85°18′3.83″E	1284	2014-10-13	23.7 °C	4 m/s	239 mm	Urban-traffic area
KTM02	Satdobato chowk	27°39′32.02″N 85°19′28.96″E	1333	2014-10-13	23.7 °C	4 m/s	239 mm	Urban-traffic area
KTM03	Koteshwor	27°40′43.41″N 85°20′55.70″E	1310	2014-10-13	23.7 °C	4 m/s	239 mm	urban -heavy traffic area
KTM04	Baneshwor	27°41′20.36″N 85°20′10.28″E	1308	2014-10-13	23.7 °C	4 m/s	239 mm	Urban-commercial area
KTM05	Mahrajganj	27°44′2.16″N 85°19′48.28″E	1329	2014-10-13	23.7 °C	4 m/s	239 mm	Urban-traffic area
KTM06	Swayambhu	27°42′56.72″N 85°17′1.71″E	1342	2014-10-13	23.7 °C	4 m/s	239 mm	Urban-traffic area
KTM07	Bhimsengola	27°42′4.67″N 85°20′24.68″E	1322	2014-10-13	23.7 °C	4 m/s	239 mm	Urban-commercial area
KTM08	Pashupati	27°42′38.74″N 85°20′46″E	1320	2014-10-13	23.7 °C	4 m/s	239 mm	Hindu pilgrim place
KTM09	Balkumari bridge	27°40′23.36″N 85°20′30.92″E	1291	2014-10-13	23.7 °C	4 m/s	239 mm	Urban-residential area
KTM10	Airport	27°42′2.97″N 85°21′18.13″E	1327	2014-10-13	23.7 °C	4 m/s	239 mm	airport
KTM11	Tinkune	27°41′7.65″N 85°20′55.13″E	1295	2014-10-13	23.7 °C	4 m/s	239 mm	urban -heavy traffic area
KTM12	Kalimati	27°41′54.88″N 85°17′52.76″E	1300	2014-10-13	23.7 °C	4 m/s	239 mm	Urban-commercial area
KTM13	Kalanki	27°41′36.44″N 85°16′51.37″E	1316	2014-10-14	23.7 °C	4 m/s	239 mm	urban -heavy traffic area
KTM14	Sinamangal	27°41′46.46″N 85°21′01.35″E	1300	2014-10-14	23.7 °C	4 m/s	239 mm	Urban -proximity to the airport
KTM15	Balazu Industrial area	27°43′48.17″N 85°18′03.82″E	1299	2014-10-14	23.7 °C	4 m/s	239 mm	Urban-industrial area
KTM16	Bagbazar	27° 42′ 22.96″N 85° 19′ 08.66″E	1297	2014-10-14	23.7 °C	4 m/s	239 mm	Urban-commercial area
KTM17	Dhapasi height	27° 44′ 58.87″N 85° 19′ 54.12″E	1347	2014-10-14	23.7 °C	4 m/s	239 mm	Urban-residential area
KTM18	Gwarko, Ring Road	27° 39′ 58.62″N 85° 19′ 58.79″ E	1299	2014-10-14	23.7 °C	4 m/s	23 9mm	Urban-traffic area
KTM19	Srijana Nagar	27° 39′ 57.17″N 85° 23′ 58.75″ E	1351	2014-10-14	23.7 °C	4 m/s	239 mm	Suburban-residential area

**Table 2:** Details about sediment sampling location.

Sample ID	Sampling site location	Lat & Long	Sampling period
BGS01	Gokarna	27° 43′ 57.66″N 85° 23′ 7.45″E	2014-10-16
BGS02	Guheshwori	27° 42′ 42.32″N 85° 21′ 13.47″E	2014-10-16
BGS03	Gaurighat	27° 42′ 46.87″N 85° 20′ 59.75″E	2014-10-16
BGS04	Pashupati	27° 42′ 35.12″N 85° 20′ 55.43″E	2014-10-16
BGS05	Tilganga	27° 42′ 12.74″N 85° 20′ 59.87″E	2014-10-16
BGS06	Sinamangal	27° 41′ 56.01″N 85° 20′ 48.24″E	2014-10-16
BGS07	Jagriti Nagar	27° 41′ 32.84″N 85° 21′ 4.83″E	2014-10-16
BGS08	Gairigaon	27° 41′ 18.40″N 85° 20′ 53.61″E	2014-10-16
BGS09	Tinkune	27° 41′ 10.42″N 85° 20′ 37.34″E	2014-10-16
BGS10	Sahyogi Nagar	27° 40′ 57.79″N 85° 20′ 21.15″E	2014-10-16
BGS11	Chhitij Nagar	27° 40′ 44.47″N 85° 20′ 4.23″E	2014-10-17
BGS12	Shankhmul	27° 40′ 50.28″N 85° 19′ 48.37″E	2014-10-17
BGS13	Jwagal	27° 41′ 10.29″N 85° 19′ 35.14″E	2014-10-17
BGS14	Thapathali	27° 41′ 22.27″N 85° 18′ 59.37″E	2014-10-17
BGS15	Tirpureshwor	27° 41′ 31.75″N 85° 18′ 37.38″E	2014-10-17
BGS16	Sanepa	27° 41′ 34.26″N 85° 18′ 17.52″E	2014-10-17
BGS17	Teku Dovan	27° 41′ 27.94″N 85° 18′ 7.61″E	2014-10-17
BGS18	Balkhu	27° 41′ 3.99″N 85° 17′ 58.17″E	2014-10-17
BGS19	Sundarighat	27° 40′ 28.29″N 85° 17′ 36.35″E	2014-10-17
BGS20	Chobhar	27° 39′ 28.46″N 85° 17′ 37.04″E	2014-10-17

**Table 3:** Full name and GS-MS parameter of OPEs.

Acronym	Full name	CAS No.	Chemical formula	Mol. Wt.	Quantifier/Qualifier	RT
TNBP	Tri-*n*-butyl phosphate	126-73-8	C_12_H_27_O_4_P	266.3	155/99	7.063
TCEP	Tris (2-chloroethyl)phosphate	115-96-8	C_6_H_12_Cl_3_O_4_P	285.5	249/143	7.696
TCIPP-1	Tris (1-chloro-2-propyl) phosphate (mix of three isomers)	13674-84-5	C_9_H_18_Cl_3_O_4_P	327.6	125/277	7.877
TCIPP-2					125/277	7.952
TCIPP-3					125/277	8.022
TDCIPP	Tris (1,3-dichloropropyl) phosphate	13674-87-8	C_9_H_15_Cl_6_O_4_P	430.9	191/381	12.210
TPHP	Triphenyl phosphate	115-86-6	C_18_H_15_O_4_P	326.3	170/228	13.107
EHDPHP	2-Ethylhexyl diphenyl phosphate	1241-94-7	C_20_H_27_O_4_P	362.4	251/170	13.329
TEHP	Tri (2-Ethylhexyl)phosphate	78-42-2	C_24_H_51_O_4_P	434.6	113/211	13.592
TMPP-1	Tri-cresyl phosphate (mix of three isomers)	1330-78-5	C_21_H_21_O_4_P	368.4	243/170	16.020
TMPP-2					243/170	16.400
TMPP-3					243/170	16.790
TCEP-d12	deuterated tris (2-chloroethyl) phosphate	1276500-47-0	C_6_H_12_Cl_3_O_4_P	297.5	261/148	7.635
HMB	Hexamethylbenzene	87-85-4	C_12_H_18_	162.3	162/147	6.330

**Table 4:** Level of average OPEs and RSD detected in blank samples of soil and sediments.

OPEs	Soil blank (ng/g)		Sediments blank (ng/g)	
	Lab (n = 10)	RSD (%)	Lab (n = 10)	RSD (%)
TNBP	4.80	1.9	4.68	1.40
TCEP	3.54	0.27	3.36	1.10
TCIPPs	4.69	0.65	4.15	0.82
TDCIPP	0.48	0.01	5.84	1.48
TPHP	5.12	2.60	1.30	0.51
EHDPHP	9.10	2.66	1.47	0.23
TEHP	0.82	0.41	ND	0
TMPPs	2.17	0.16	4.35	1.40

**Table 5:** Concentration of OPEs in surface soil from Kathmandu valley (ng/g).

Sampling sites	OPE compounds							
	TNBP	TCEP	TCIPPs	TDCIP	TPHP	EHDPHP	TEHP	TMPPs
KTS-1	16.15	9.50	165.24	29.28	67.66	51.21	27.54	101.65
KTS-2	17.05	2.40	77.73	14.54	5.06	25.57	26.53	38.31
KTS-3	14.63	2.29	22.41	22.45	12.57	19.79	20.83	40.21
KTS-4	17.40	2.42	31.65	0.65	11.10	27.52	29.65	84.92
KTS-5	14.59	1.04	17.52	20.20	9.61	20.02	18.85	21.53
KTS-6	14.91	0.72	11.23	19.07	8.10	18.92	19.56	21.05
KTS-7	17.74	13.27	31.13	0.79	3.14	25.64	26.05	27.58
KTS-8	16.95	12.65	30.82	0.75	5.49	25.10	25.87	41.33
KTS-9	17.52	9.96	37.77	0.33	3.15	29.34	24.89	31.40
KTS-10	14.90	3.03	20.41	19.46	12.94	19.81	19.88	16.99
KTS-11	14.35	27.02	33.87	19.11	14.20	23.34	16.79	27.42
KTS-12	18.45	27.11	18.10	19.49	5.40	10.22	27.63	30.03
KTS-13	17.16	22.86	20.22	12.57	24.31	34.82	21.26	125.93
KTS-14	15.52	18.37	19.79	12.33	17.65	20.78	8.52	114.65
KTS-15	53.25	83.67	990.52	14.09	39.39	59.77	239.41	230.04
KTS-16	16.52	29.45	49.57	43.87	86.85	113.65	94.78	1312.56
KTS-17	27.92	25.29	252.37	64.76	54.87	42.86	221.71	697.59
KTS-18	13.53	75.21	386.22	60.43	49.39	101.37	858.06	25280.46
KTS-19	16.15	9.50	165.24	29.28	67.66	51.21	27.54	101.65

**Table 6:** Concentration of OPEs in sediment samples (ng/g).

Sampling sites	OPE compounds							
	TNBP	TCEP	TCIPPs	TDCIP	TPHP	EHDPHP	TEHP	TMPPs
BGS-1	5.04	16.04	54.49	4.25	47.30	117.08	1149.72	945.30
BGS-2	10.23	10.96	14.67	3.89	7.92	52.11	2379.19	431.07
BGS-3	25.62	16.31	12.25	4.75	20.27	170.22	978.52	462.78
BGS-4	40.46	18.78	26.14	3.99	16.98	417.60	1151.87	982.32
BGS-5	11.24	11.26	1.69	5.53	3.33	37.71	1639.89	509.45
BGS-6	16.87	12.94	8.71	8.93	13.06	36.74	1699.33	800.55
BGS-7	46.89	24.11	84.35	6.32	39.19	148.92	1004.35	2162.37
BGS-8	57.40	19.90	883.48	4.98	32.05	179.12	1091.76	938.72
BGS-9	78.40	35.39	90.70	6.62	91.40	164.93	778.17	459.15
BGS-10	93.48	38.30	51.00	6.14	130.09	196.37	777.60	469.84
BGS-11	29.46	17.01	7.84	5.21	15.05	99.84	1713.72	1038.28
BGS-12	29.83	18.60	16.84	5.96	21.16	132.16	3024.98	1334.29
BGS-13	239.25	17.98	218.55	8.54	129.46	158.51	703.86	1306.01
BGS-14	319.93	12.81	333.32	5.41	12.35	238.99	656.50	1046.58
BGS-15	17.48	12.83	5.23	4.97	7.97	47.84	1970.74	548.77
BGS-16	39.50	16.79	22.74	4.96	19.37	58.66	2292.37	481.95
BGS-17	213.23	14.61	152.45	7.45	51.71	117.72	886.32	1510.93
BGS-18	183.27	11.97	196.61	6.64	19.76	130.33	704.13	1074.47
BGS-19	16.41	13.67	15.73	4.10	5.24	33.93	1364.76	284.58
BGS-20	54.26	28.91	55.00	4.47	108.19	173.93	1136.40	1629.74

**Table 7:** Health risk exposure of OPE via soil ingestion and dermal contact (ng/kg bw/day).

OPE	Soil ingestion	Dermal absorption via soil	RfD
TNBP	0.005	0.040	2.4 × 10^4^
TCEP	0.004	0.031	2.2 × 10^4^
TCIPPs	0.009	0.077	8.0 × 10^4^
TDCIP	0.005	0.046	1.5 × 10^4^
TPHP	0.004	0.031	7.0 × 10^4^
EHDPHP	0.007	0.062	–
TEHP	0.007	0.063	–
TMPPs	0.012	0.100	1.3 × 10^4^
∑OPE	0.053	0.451

**Table 8:** Estimated Risk quotient (RQ) of different OPEs for fish and total RQ in the Bagmati River.

Fish	TNBP	TCEP	TCIPPs	TDCIP	TPHP	TMPPs	∑OPE
BGS-1	0.4	0.2	1.4	1.9	55.4	3562.4	3621.7
BGS-2	0.8	0.1	0.4	1.8	9.4	1639.1	1651.6
BGS-3	11.7	0.9	1.8	12.5	138.9	10197.9	10363.7
BGS-4	21.2	1.2	4.4	12.0	132.9	24730.1	24901.7
BGS-5	0.6	0.1	0.0	1.6	2.5	1217.9	1222.6
BGS-6	0.9	0.1	0.1	2.6	9.9	1955.1	1968.7
BGS-7	2.7	0.2	1.6	2.1	34.4	6100.6	6141.6
BGS-8	3.1	0.1	15.6	1.6	26.3	2476.6	2523.3
BGS-9	2.9	0.2	1.1	1.4	51.1	826.2	883.0
BGS-10	3.9	0.2	0.7	1.5	82.0	952.8	1041.2
BGS-11	5.7	0.4	0.5	5.8	43.5	9646.9	9702.8
BGS-12	6.7	0.5	1.2	7.7	71.2	14430.8	14518.1
BGS-13	7.0	0.1	2.0	1.4	56.4	1828.8	1895.7
BGS-14	16.8	0.1	5.6	1.6	9.7	2645.5	2679.4
BGS-15	2.3	0.2	0.2	3.8	15.9	3511.1	3533.5
BGS-16	5.1	0.3	0.9	3.7	37.2	2973.7	3020.8
BGS-17	6.5	0.1	1.5	1.3	23.6	2213.8	2246.7
BGS-18	9.4	0.1	3.2	2.0	15.2	2658.8	2688.7
BGS-19	2.7	0.3	0.8	3.9	13.1	2286.9	2307.7
BGS-20	10.8	0.7	3.5	5.1	321.5	15577.6	15919.2

**Table 9:** Estimated Risk quotient (RQ) of different OPEs for Daphnia and total RQ in the Bagmati River.

Daphnia	TNBP	TCEP	TCIPPs	TDCIP	TPHP	TMPPs	∑OPE
BGS-1	1.5	0.0	0.5	0.5	21.2	1451.4	1475.1
BGS-2	3.1	0.0	0.1	0.5	3.6	667.8	675.2
BGS-3	45.6	0.2	0.6	3.6	53.0	4154.7	4257.7
BGS-4	82.2	0.3	1.4	3.4	50.7	10075.2	10213.3
BGS-5	2.2	0.0	0.0	0.5	0.9	496.2	499.8
BGS-6	3.3	0.0	0.0	0.7	3.8	796.5	804.4
BGS-7	10.7	0.0	0.5	0.6	13.1	2485.4	2510.4
BGS-8	12.2	0.0	5.1	0.4	10.0	1009.0	1036.9
BGS-9	11.4	0.0	0.4	0.4	19.5	336.6	368.3
BGS-10	15.3	0.1	0.2	0.4	31.3	388.2	435.5
BGS-11	22.1	0.1	0.2	1.7	16.6	3930.2	3970.8
BGS-12	26.0	0.1	0.4	2.2	27.2	5879.2	5935.1
BGS-13	27.0	0.0	0.7	0.4	21.5	745.1	794.7
BGS-14	65.3	0.0	1.9	0.5	3.7	1077.8	1149.1
BGS-15	9.0	0.1	0.1	1.1	6.1	1430.4	1446.7
BGS-16	19.7	0.1	0.3	1.0	14.2	1211.5	1246.8
BGS-17	25.2	0.0	0.5	0.4	9.0	901.9	937.0
BGS-18	36.6	0.0	1.1	0.6	5.8	1083.2	1127.2
BGS-19	10.6	0.1	0.3	1.1	5.0	931.7	948.8
BGS-20	41.9	0.2	1.2	1.5	122.8	6346.4	6513.8

**Table 10:** Estimated Risk quotient (RQ) of different OPEs for algae and total RQ in Bagmati River.

Algae	TNBP	TCEP	TCIPPs	TDCIP	TPHP	TMPPs	∑OPE
BGS-1	0.6	0.3	0.9	0.1	46.6	1351.3	1399.7
BGS-2	1.3	0.2	0.2	0.1	7.9	621.7	631.4
BGS-3	18.4	1.6	1.2	0.4	116.7	3868.2	4006.4
BGS-4	33.3	2.1	2.9	0.4	111.6	9380.4	9530.6
BGS-5	0.9	0.1	0.0	0.0	2.1	462.0	465.1
BGS-6	1.3	0.1	0.1	0.1	8.3	741.6	751.6
BGS-7	4.3	0.3	1.1	0.1	28.9	2314.0	2348.6
BGS-8	4.9	0.2	10.4	0.0	22.1	939.4	977.1
BGS-9	4.6	0.3	0.7	0.0	43.0	313.4	362.0
BGS-10	6.2	0.3	0.5	0.0	68.9	361.4	437.4
BGS-11	8.9	0.7	0.3	0.2	36.5	3659.2	3705.8
BGS-12	10.5	0.9	0.8	0.2	59.8	5473.7	5546.0
BGS-13	10.9	0.1	1.4	0.0	47.3	693.7	753.5
BGS-14	26.4	0.1	3.7	0.1	8.2	1003.5	1042.0
BGS-15	3.7	0.4	0.1	0.1	13.3	1331.8	1349.4
BGS-16	8.0	0.5	0.6	0.1	31.2	1128.0	1168.3
BGS-17	10.2	0.1	1.0	0.0	19.8	839.7	870.8
BGS-18	14.8	0.1	2.2	0.1	12.8	1008.5	1038.4
BGS-19	4.3	0.5	0.6	0.1	11.0	867.4	883.9
BGS-20	16.9	1.2	2.3	0.2	270.1	5908.7	6199.5

## 2 Experimental design, materials, and methods

### 2.1 Soil sampling

A total of 19 surface soil (0–15 cm depth, vegetation removed) samples were collected at 19 different locations in Kathmandu Valley using stainless steel scoops during 15–25 October 2014 (Table 1). Each soil sample was a composite of 3 sub-samples, which was collected in a radius of 5 m in a different direction. The soil samples were then wrapped in an aluminum foil packed into sealed polythene bags and kept in an ice bag before transporting to the laboratory. Hand gloves were used to avoid contamination during sampling. The soil samples were freeze-dried, ground to powder and sieved through 500 μm sieve and stored at −20 °C until chemical analysis.

### 2.2 Sediments sampling

**Fig. 1:**
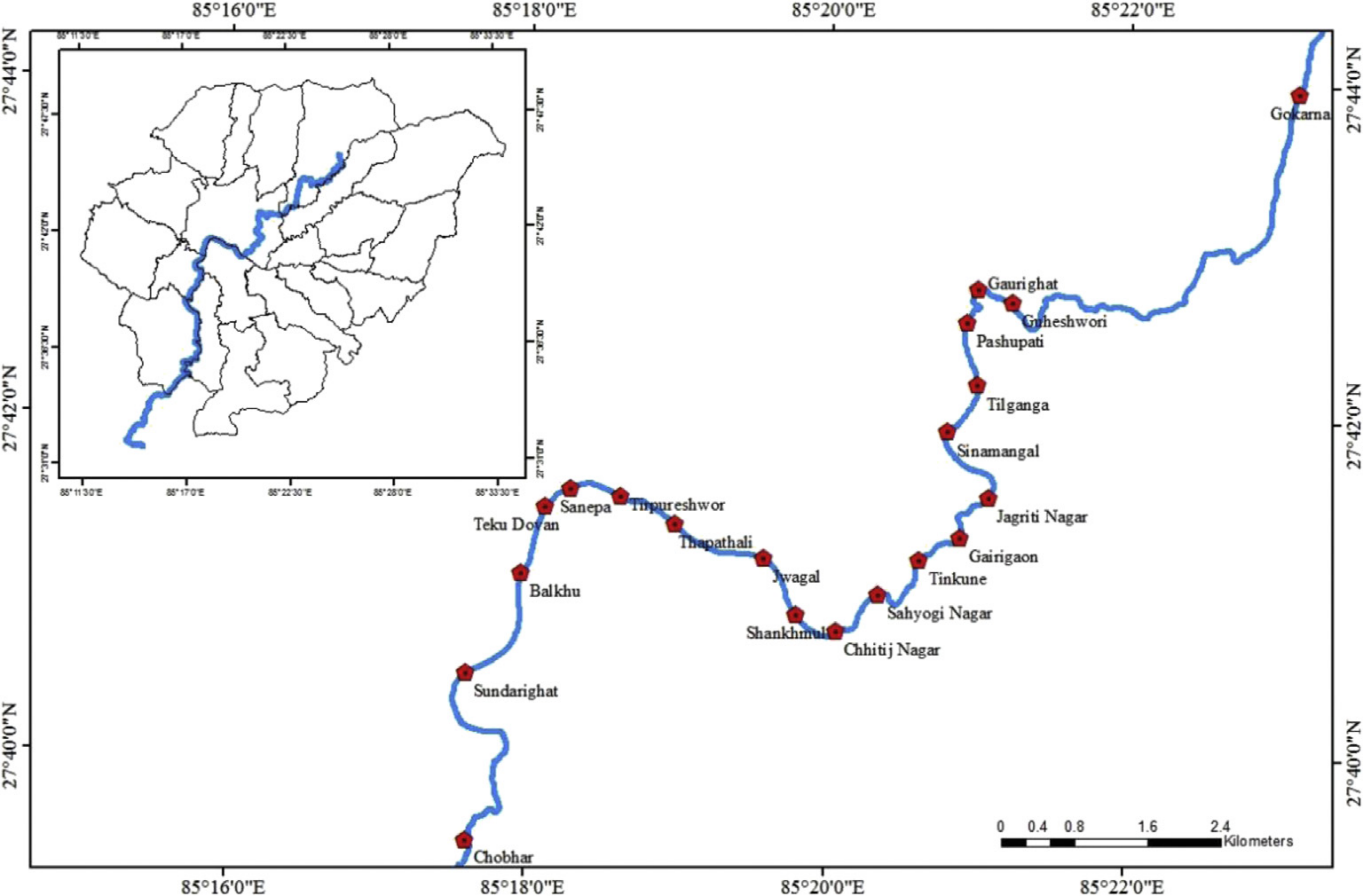
Map of Bagmati river in Kathmandu showing sediment sampling point.

Bagmati River, which flows through the capital city Kathmandu was chosen for the collection of sediment samples. About 50g of surface sediment (top 5 cm) samples were collected using pre-cleaned stainless steel scoop at 20 sites along Bagmati River (a stretch of > 27 km), from Gokarneshwor in the north to Chobhar in the south. Fig. 1 and Table 2 shows the sampling points along the Bagmati River. Different items, like rocks, sticks, mussels, etc. Were removed from the sediment samples and transported to the laboratory keeping in an icebox. Later, all the sediment samples were stored in the refrigerator at −20 °C until chemical analysis. The sediment samples were freeze-dried, ground to fine, sieved through mesh size of 500 μm, and kept in an amber jar until extraction.

**Fig. 2:**
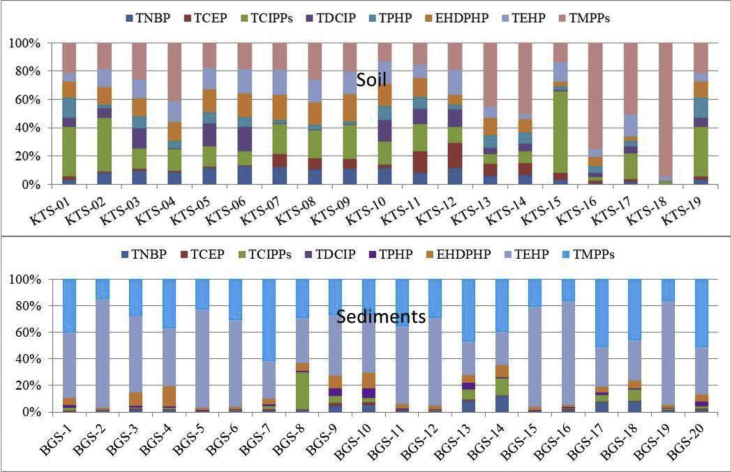
Site-specific profile of OPE compounds in soil (top) and sediments (bottom) from Kathmandu (adopted from Yadav et al., 2018).

**Fig. 3:**
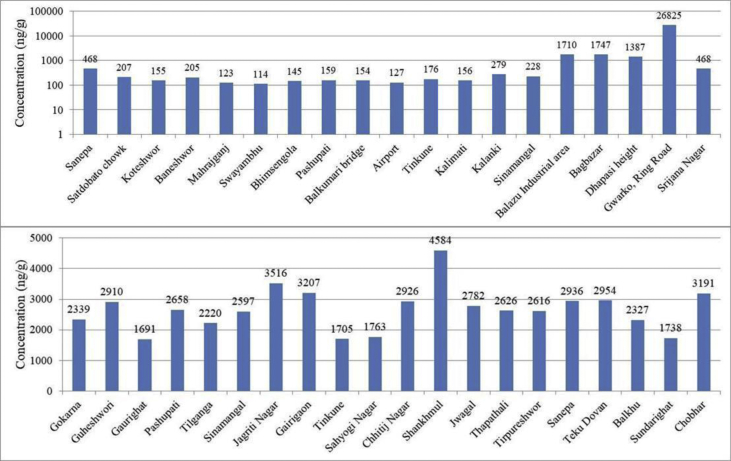
Spatial distribution of OPE in surface soil (top) and sediment (bottom) from Kathmandu.

### 2.3 Sample preparation and analysis

Freeze-dried and homogenized soil/sediment samples were spiked with 1000 ng of deuterated tris (2-chloroethyl) phosphate (TCEP-d12) as a recovery standard. Later, they were Soxhlet extracted with dichloromethane (DCM) for 24 h. Copper granules were added to the round bottle flask before extraction to remove the elemental Sulphur present in soils and sediments. Copper granules were prewashed and activated with hydrochloric acid before adding to the container. The sample extract was concentrated by rotary evaporator (Heildolph 4000, Germany) and was solvent exchanged to hexane with a volume of 0.5 mL. The extract was passed through Supelclean Envi Florisil SPE column tubes 6 mL (1g) (SUPELCO, USA) for purification. Before fractionation, Florisil® cartridges were prewashed with 6 mL ethyl acetate, 6 mL hexane/DCM (8:2, v/v), and 10 mL hexane to clean and condition the adsorbent. After the extract was transferred to the SPE column, the first fraction was eluted with 6 mL 8:2 Hex: DCM and was discarded. The second fraction that contained target OPFRs were eluted with 20ml ethyl acetate, evaporated until dryness under constant nitrogen flow and the residue was re-dissolved in 200 μL of iso-octane. The resulting fraction was transferred to GC vials for GC-MS analysis. Before GC-MS injection, a known amount (1000 ng) of hexamethyl benzene (HMB) was added as an internal standard for quantification purposes.

### 2.4 GC-MS analysis

Eight target OPEs (TCEP, TCIPPs: mix of three isomers, TDCIPP, TNBP, TEHP, TPHP, EHDPHP and TMPPs: mix of three isomers) were analyzed using Agilent GC (7890A) coupled with 7000A Triple quadrupole coupled MSD, with a DB5-MS capillary column (30 m × 0.25 mm i. d. × 0.25 μm film thickness). One μL of the sample was injected in splitless mode, and the temperature of the injector was 295 °C. Helium was used as carrier gas at the flow rate of 1 mL min-^1^. The temperature of the transfer line and ion source was maintained at 280 °C and 230 °C, respectively. The GC oven temperature started at 60 °C for 1 min, increased to 220 °C at a rate of 30 °C min^−1^ (held for 0 min), then to 300 °C at a rate of 5 °C min^−1^ (held for 15 min). The full name of eight OPEs is given in Table 3.

### 2.5 Quality assurance/quality control

Since OPFRs are ubiquitous to the indoor environment, we adopted strict precaution and QA/QC criteria to minimize the contamination. All the glassware was soaked in 5% KOH and 95% ethanol solution and washed with Milli-Q water, followed by DCM and hexane. Then the cleaned glassware was oven-dried and was baked at 450 °C for 6h and rinsing with solvents. Although we took utmost care, it appears that contamination with OPEs may occur at some point in extraction, clean up, or analysis in the laboratory. Hence, we followed a rigorous cleaning procedure before experimentation to ensure minimum contamination of OPEs. We used prebaked Na_2_SO_4_ as a blank soil/sediment sample, which was packed in aluminum foil and taken to sampling sites and brought back to the laboratory with soil/sediment samples.

Ten laboratories blank each for soil, and sediments were extracted and analyzed together with samples to assess the possible contamination of the samples. The level of OPFRs detected in laboratory blank ranged from 0.48 to 9.10 ng/g and 1.30–5.84ng/g for soil and sediments, respectively (Table 4). The method detection limits (MDLs) are the mean plus three times the standard deviation of all the blank samples. When the compounds were not detected in the blank, the MDL was calculated as three times signal to noise ratio obtained from the lowest spiked standard. The MDLs of OPFRs ranged from 0.51 to 17.08 ng/g and 2.83–17.52 ng/g in soil and sediments, respectively. The average recovery of the surrogate standard (TCEP-d12) was 108 ± 6.4% and 124 ± 5.2% for soil and sediments, respectively. In this data, the concentrations of target OPFRs were blank corrected, but not corrected for recovery.

### 2.6 Health risk assessment

Soil ingestion and dermal absorption of soil to the general population of Kathmandu were assessed using the health risk assessment model recommended by USEPA and is expressed in the following equations (1), (2). All the constant factors/parameters used in the health risk assessment model were obtained from the literature [[2], [3], [4]].

(1) $$Soil\;ingestion\;exposure=\frac{CS\times DI}{BW}$$

(2) $$Dermal\;contact\;via\;soil=\frac{CS\times DAS\times ESA\times AF}{BW}$$

where C_S_ refers level of OPEs in soil (ng/g), DI is daily soil intake (20 mg/day) [2]. DAS represents dust adhered to skin rate (0.01mg/cm^2^) [[2], [3], [4]], while ESA denotes exposed skin area (1000cm^2^) [2]. AF signifies absorption factor (0.17%) [5].

### 2.7 Ecological risk assessment

The risk quotient (RQ) approach is widely used to assess the impact of pollutants on non-target aquatic organisms. In this data, sediment-based OPE concentration is utilized to determine the risk of OPE contaminants on aquatic organism recommended by Santos et al. [6] and Sanchez-Avila et al. [7]. The RQs are estimated as a quotient of the observed environmental level and the predicted no-effect concentration.

(3) $$RQ=\frac{MEC}{PNEC}$$

(4) $$PNEC=\frac{EC_{50}}{f}$$

where MEC refers to the measured environmental concentration. PNEC denotes the predicted no-effect level and can be calculated as the ratio of the toxicological relevant concentration (EC_50_) and security factor (*f*). The EC_50_ for OPE compounds was acquired from the Environmental Risk Limits report for OPEs [8], while 1000 was used as a security factor. The risk was estimated exclusively for those chemicals where EC_50_ is available. In this data, MEC was representing pore water concentration and used instead of the direct measure of OPEs in sediments. We assumed that pore-water is the main route of exposure for fish, Daphnia, and alga. The pore-water concentration was estimated using the equilibrium partitioning approach suggested by Di Toro et al. [9].

(5) $$C_{pw}=\frac{C_{s}}{f_{oc}K_{oc}}$$

where C_*pw*_ represents calculated pore water concentration, C_s_ is the concentration of OPEs in sediments (mg/kg), *f*_oc_ denotes the content of organic carbon in sediments, and *K*_oc_ is the partitioning coefficient organic carbon in sediment. The degree of ecological risk due to sediment contamination can be estimated based on the magnitude of RQ. The RQ value < 1, denote no significant risk, RQ between 1 and < 10, indicates the small potential for adverse effect, RQ between 10 and <100, specifies considerable potential for adverse effect, and RQ ≥ 100, suggests potential adverse effect expected.
